# Effect of Side Chain Length on Polycarboxylate Superplasticizer in Aqueous Solution: A Computational Study

**DOI:** 10.3390/polym11020346

**Published:** 2019-02-17

**Authors:** Po-Hsiang Chuang, Yu-Hui Tseng, Yunhui Fang, Miaomiao Gui, Xiuxing Ma, Jinjing Luo

**Affiliations:** 1 Xiamen Academy of Building Research Group Co., Ltd., Xiamen 361004, China; mututu@126.com (M.G.); mary@xmabr-kzj.com (X.M.); 2 KZJ New Materials Group Co., Ltd., Xiamen 361101, China; fangyunhui@126.com; 3 College of the Environment & Ecology, Xiamen University, Xiamen 361102, China; luojj27@xmu.edu.cn; 4 College of Chemistry and Environment, Minnan Normal University, Zhangzhou 363000, China; tsenglittleyuyu@gmail.com

**Keywords:** molecular dynamics simulation, polycarboxylate superplasticizer, solution conformation, side chain effect

## Abstract

Molecular dynamics simulations were carried out to study the conformations of polycarboxylate ether (PCE) superplasticizers with different side chain lengths in aqueous solution. For four types of PCE molecules—PCE1, PCE2, PCE3, and PCE4—the steric hindrance between the PCE molecules increased with increasing side chain length. The side chain length not only affects water mobility but also affects the distribution of water molecules in the system. Simulation results indicate that water molecules were trapped by the PCE molecules, reducing the diffusion properties. PCE molecules with long side chains have more water molecules probability around the main chain and fewer water molecules probability near the side chain. Microscopic-level knowledge of the interaction between superplasticizer and water molecules facilitates understanding of the performance of superplasticizers in cement systems.

## 1. Introduction

In recent years, different types of chemical admixtures have attracted increasing attention from the cement industry [1,2,3,4]. Superplasticizers or high-range water reducers are one kind of chemical admixtures that are added to cement mixtures to improve the workability of cement and concrete [5,6]. Polycarboxylate ether (PCE) superplasticizers are easily designed and synthesized for different functions and situations [2,7,8,9,10,11]. PCEs are usually composed of backbones containing anionic groups and side chains designed from different types of polyoxyethylene glycols (PEGs) [12]. The anionic groups electrostatically adsorb onto the cement surface and the side chains provide steric repulsion, resulting in excellent cement dispersing effect.

The side chain length of a superplasticizer is a key factor affecting steric hindrance. Many researchers have suggested that the steric hindrance resulting from the PCE side chain on the particle surfaces affecting the dispersion behavior in the system [9,13,14,15]. These studies showed the dispersion performance of PCE in the systems by various experiments. Yamada et al. reported that PCE with longer side chains have higher dispersing properties in cement paste [15]. Ran et al. synthesized a series of PCEs with different side chain lengths to investigate the dispersing mechanism in terms of rheological behavior and dispersion in cement system [9]. Winnefeld et al. investigated PCEs with polyether chains of varying lengths and densities using various characterization methods [16]. The dispersion properties of PCE varies depending on the side chain length in the polymer.

Recently, computational simulation has been widely applied in studying various systems [17,18,19]. Computer simulation is rapidly becoming a standard tool for studying the structure and dynamics of polymers by investigating molecular interactions at the microscopic level. Molecular dynamics (MD) simulations have been used to elucidate the working mechanism of PCEs [20]. Zhao et al. investigated the adsorption conformations of hydrophobically modified PCE on dicalcium silicate surface using all-atom MD simulations [21]. Hirata et al. simulated the PCE polymers adsorbed on an MgO surface in cement pore solutions [22]. However, deeper insights at the atomic or molecular level are necessary to achieve better bottom-up understanding and more effective performance control of superplasticizers. 

In this study, we investigated four PCE molecules with side chains of different lengths in aqueous solution using all-atom MD simulations. In cement suspension systems, PCE not only adsorbs on the cement particles, but also self-aggregates in the dispersed system [23,24]. The interaction between PCE and water molecules is the main factor affecting the properties of superplasticizers. The effects of the side chain length of PCE were analyzed and observed in terms of intermolecular interactions at atomic level. The results of this study facilitate better understanding of the dispersion mechanism and properties of PCE molecules in cement systems.

## 2. Methods 

**Figure 1:**
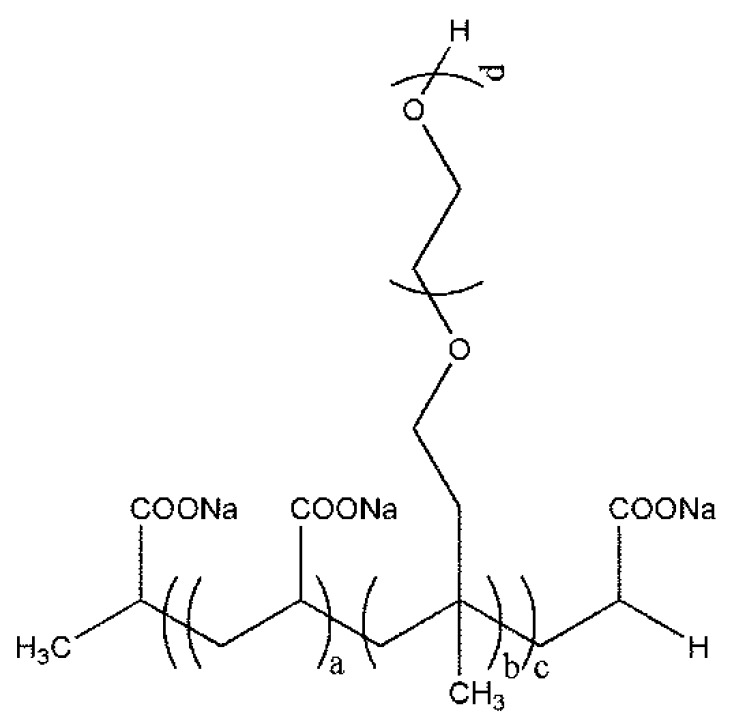
The chemical structure of polycarboxylate ether PCE.

All-atom MD simulations were conducted to study the solution conformation properties of PCEs with different side chain lengths. Figure 1 shows the chemical structure of PCE. The backbone of PCE may be regarded as being composed of two kinds of repeated units—acrylic acid (AA) sodium salt and isopentenyl polyethylene glycol (TPEG)—with the acid-to-ether molar ratio (a:b) being 4:1. The side chains were equally spaced on the backbone and the number of side chains (c) is 10. Four PCE molecules—PCE1, PCE2, PCE3, and PCE4—with different side chain lengths were designed and the degrees of polymerization (d) of these side chains were set at 12, 26, 53, and 67, respectively, corresponding to TPEGs with molecular weights of ~600, 1200, 2400, and 3000. These TPEGs are also consistent with the commonly used experimental samples. The molecular weights of PCE range from 9000 to 33,000, which is consistent with the molecular weight of our synthesized PCE polymer.

All MD simulations were carried out in the canonical ensemble (NVT). The temperature of the system was set at 298 K and the time step of integration was set at 1.0 fs. The PCE and 3000 water molecules were randomly placed in the system with periodic boundary conditions. All the systems eventually reached thermodynamic equilibrium by checking the radius of gyration (Rg) of PCE molecules. The COMPASS (condensed-phase optimized molecular potentials for atomistic simulation studies) force field was used [25], which has the necessary parameters for all the atoms in the periodic table. The COMPASS force field is an excellent potential model suitable for the simulations of different types of common organic molecules, polymers, metal halides, silica/aluminosilicates, and metal oxides. For long-range interactions the Ewald method was used with the cutoff radius of 6 Å [26]. Simulations were performed using the Accelrys Materials Studio 6.0 package (Accelrys commercial software, Dassault Systèmes BIOVIA, San Diego, CA, USA).

## 3. Results and Discussion

### 3.1. The Conformation Properties of PCE Molecules in Water Solution

**Figure 2:**
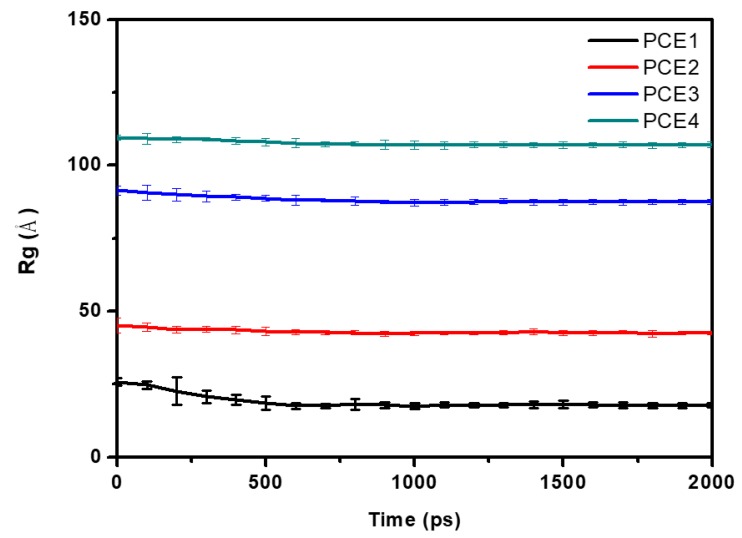
The radius of gyration (Rg) of PCE molecules in systems.

**Figure 3:**
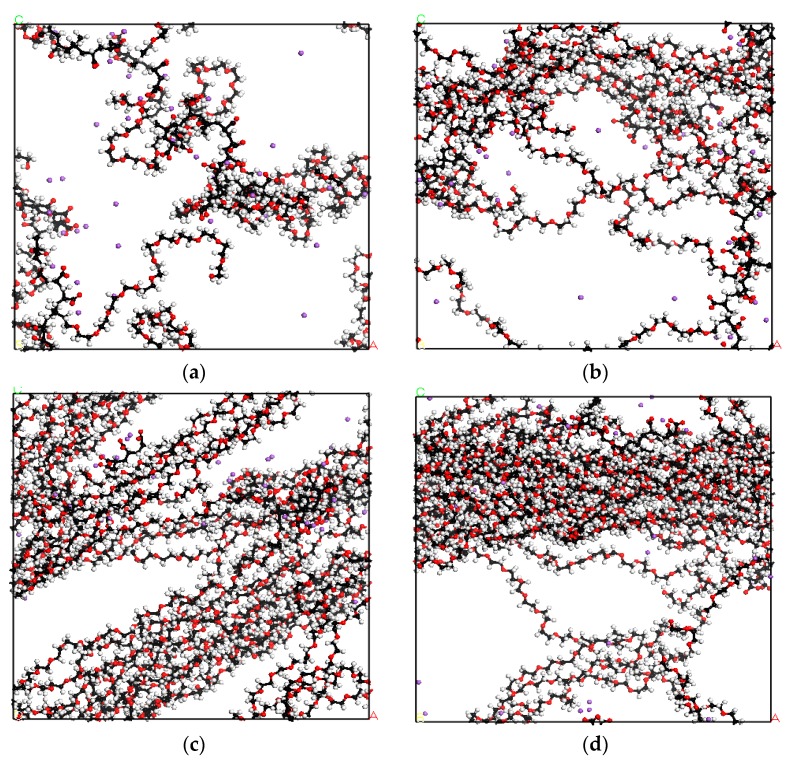
The snapshots of (**a**) PCE1, (**b**) PCE2, (**c**) PCE3, and (**d**) PCE4 in aqueous systems. The black, red, and white balls represent carbon, oxygen, and hydrogen atoms, respectively.

Using MD simulations, we studied the solution conformation structure of the four PCE molecules—PCE1, PCE2, PCE3, and PCE4—in an aqueous system. After equilibrium, an extra simulation for 1.0 ns was carried out to make sure the system reached thermodynamics equilibrium. Figure 2 shows the Rg of PCE molecules of the last 2.0 ns in the trajectory. Figure 3 shows the snapshots of PCE1, PCE2, PCE3, and PCE4 in the solution systems after equilibration; the water molecules were removed for clarity. Figure 3 clearly shows the effect of side chain length on the intermolecular interaction in PCE. The longer the side chain of PCE is, the greater is the aggregation between PCE molecules, resulting in increased steric repulsion of PCE molecules.

**Figure 4:**
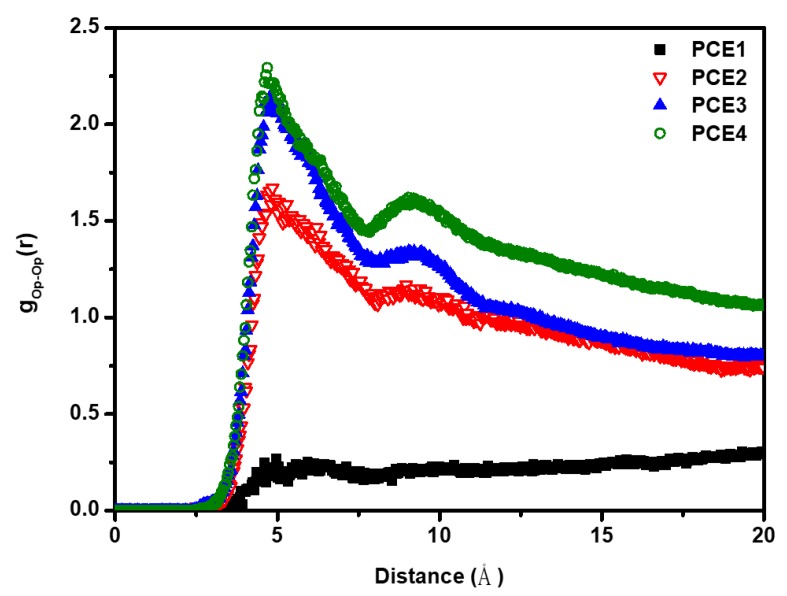
The radial distribution functions (RDFs) g_Op-Op_(r) in PCE aqueous systems.

To analyze the interaction between PCE molecules, various radial distribution functions (RDFs) were calculated. RDF shows the local structure and understanding the atomic distribution of substance, which obtains the effective intermolecular potentials and the probability between two objects [27,28]. The intermolecular relationship between PCE molecules is shown in Figure 4; Op denotes the oxygen atom on the PCE molecule. Figure 4 shows that the interaction between PCE molecules increased with increasing side chain length of PCE. The side chain of PCE molecules aggregate in a homogeneous manner, which makes the steric hindrance between PCE molecules more obvious. The intensity of the steric effect decreases in the following order, PCE4 > PCE3 > PCE2 > PCE1. The results are consistent with the observations in Figure 3.

### 3.2. The Relationship between PCE and Water Molecules in Aqueous System

Water molecules play an important role in concrete system. The water molecules affect the hydration as well as dispersion of cement. The PCE molecules as a surfactant help cement particles disperse easily in aqueous systems [29,30]. Therefore, the relationship between PCE molecules and water molecules is worth exploring. To investigate the mobility of water molecules, the mean square displacement (MSD) was calculated, and the diffusion coefficient of the particles was calculated from the slope of the MSD [31].

**Figure 5:**
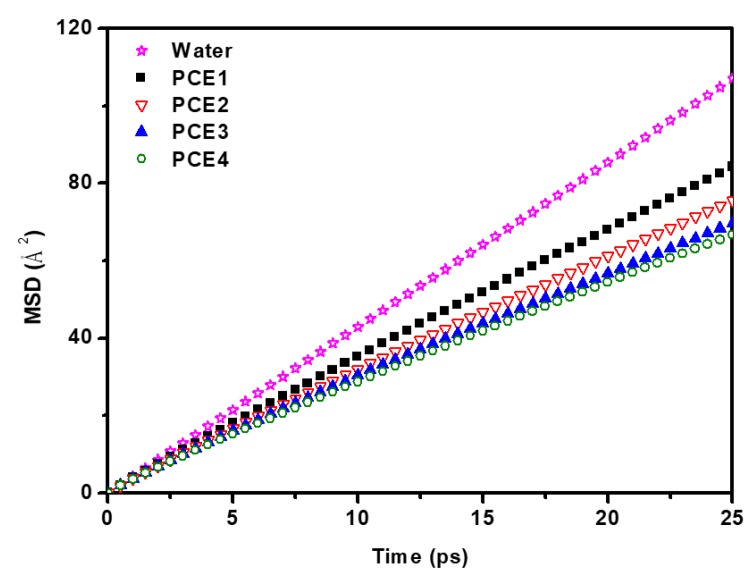
The mean square displacements (MSDs) of water molecules in different systems.

Figure 5 shows the MSDs of water molecules in different systems. When the PCE molecules were added to the system, the mobility of water molecules decreased because they were trapped by the PCE molecules. For different PCE molecules, the migration ability of water molecules decreased with increasing side chain length. The diffusion coefficients of water decreased in the following order, PCE1 > PCE2 > PCE3 > PCE4. It shows that the side chain length has stronger interaction with water molecules, and affect the mobility of the water molecules.

### 3.3. The Probability Distribution of Water Molecules

**Figure 6:**
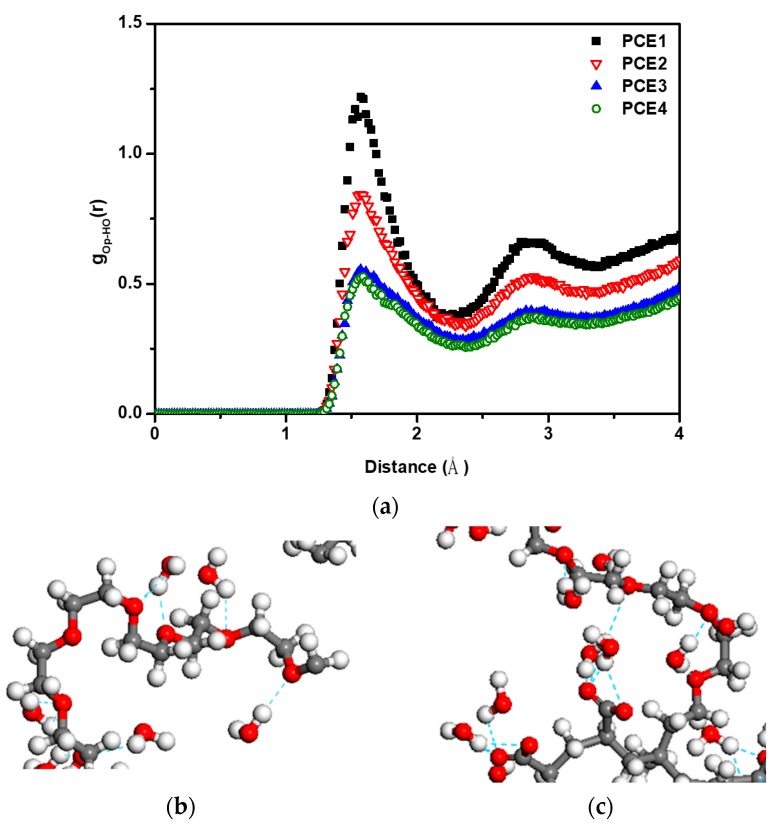
(**a**) The RDF g_Op-HO_(r) in PCE aqueous systems, (**b**) part of hydrogen bonds on the side chain of PCE, and (**c**) part of hydrogen bonds around the main chain of PCE.

**Figure 7:**
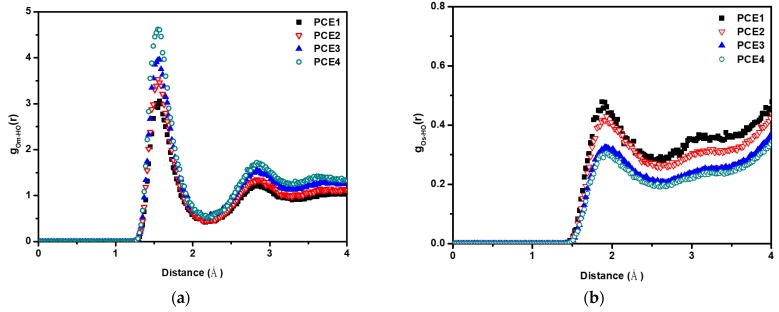
The RDFs (**a**) g_Om-HO_(r) and (**b**) g_Os-HO_(r) in PCE aqueous systems.

To understand the role of PCE and water molecules, RDFs were used to calculate a series of relationships (Figure 6 and Figure 7). Op, Om, and Os are defined as the oxygen atoms on the PCE molecule, around the main chain of PCE molecule (including main chain and the first three oxygen of side chain), and on the side chain of PCE molecule, respectively; HO is defined as the hydrogen atom on the water molecule. Figure 6a shows that all the PCE molecules have obvious hydrogen bonds with water molecules with bond distance = 1.55 Å. The snapshot of hydrogen bonds on the side chain and around the main chain is shown in Figure 6b,c. The cutoff distance and angle of hydrogen bonds are 3.0 Å and 35 degrees, respectively. Both of the side chain and the main chain form hydrogen bonds with water molecules. With increasing PCE side chain length, fewer coordination number of water molecules around the PCE molecules. To further understand the interaction between superplasticizer and water, Os and Om were separated, and the RDFs with water molecules were calculated independently, as shown in Figure 7a,b. Figure 7a shows the relationship between water and PCE molecules around the main chain of PCE. Many water molecules accumulate near the main chain of PCE molecules to form hydrogen bonds of bond length = 1.55 Å. The probability of water molecules in these four systems are PCE4 > PCE3 > PCE2 > PCE1. Figure 7b shows the situation of water molecules around the side chain of PCE molecules. The side chain of PCE molecules also have intermolecular interaction with water molecules, but the coordination number of surrounding water molecules decrease with increasing side chain length (PCE1 > PCE2 > PCE3 > PCE4).

The conformation of superplasticizers in aqueous solutions affects both the adsorbed layer on cement surface and the water density at the interface [21]. The probability distribution of water molecules around the superplasticizers vary depending on the side chain length. With increasing side chain length, the water molecules are easier surround the main chain of PCE and decreasing near the side chain as shown in Figure 7. This perturbed of the distribution of water at the surface of cement particles and influenced the hydration kinetics of cement [32]. The microscopic water-reducing performance of PCEs also increased with increasing side chain length of PCE. 

The water-reducing properties of superplasticizer in cement systems depend on van der Waals interactions, electrostatic interactions, and steric interactions [24]. These interactions contribute to the action of superplasticizer and the dispersion of cement. The intermolecular probability distribution of PCE increased with increasing side chain length, which in turn increased the steric hindrance in the systems. The composition of the water molecules surrounding the superplasticizer molecules also changed, which affected the hydration and water-reducing properties at cement surface interface. These factors all indicate that the water reducing and dispersing properties of PCE increase with increasing side chain length. The working abilities of superplasticizers are in the following order, PCE4 > PCE3 > PCE2 > PCE1, but those of PCE4 and PCE3 are similar.

## 4. Conclusions

MD simulations were conducted to investigate the effect of different side chain of PCE polymer in aqueous system. Our results showed that the steric effect intensity of PCE increased with increasing side chain length, which affected the intermolecular force of PCE polymers as well as mobility of water molecules. The water molecules were trapped around the PCE molecules, which affected the diffusion coefficient of water. All PCE molecules formed obvious hydrogen bonds with water molecules in aqueous solution. The distribution of water molecules around the PCE molecules depended on the side chain length. The longer the side chain, the coordination number of water molecules around the side chain gradually decreased, but the coordination number of water molecules around the main chain gradually increased. It was found that the side chain length of PCE affected both dispersion behavior and hydration of cement. MD simulations provided molecular insight on superplasticizers in aqueous solution, which can be useful to understand the properties of superplasticizers in cement systems at the microscopic level.

## References

[1] Uchikawa H., Sawaki D., Hanehara S. (1995). Influence of Kind and Added Timing of Organic Admixture on the Composition, Structure and Property of Fresh Cement Paste. Cem. Concr. Res..

[2] Plank J., Pöllmann K., Zouaoui N., Andres P.R., Schaefer C. (2008). Synthesis and Performance of Methacrylic Ester Based Polycarboxylate Superplasticizers Possessing Hydroxy Terminated Poly(Ethylene Glycol) Side Chains. Cem. Concr. Res..

[3] Kirby G.H., Lewis J.A. (2008). Comb Polymer Architecture Effects on the Rheological Property Evolution of Concentrated Cement Suspensions. J. Am. Ceram. Soc..

[4] Houst Y.F., Bowen P., Perche F., Kauppi A., Borget P., Galmiche L., Le Meins J.-F., Lafuma F., Flatt R.J., Schober I. (2008). Design and Function of Novel Superplasticizers for More Durable High Performance Concrete (Superplast Project). Cem. Concr. Res..

[5] Alonso M.M., Palacios M., Puertas F. (2013). Compatibility between Polycarboxylate-Based Admixtures and Blended-Cement Pastes. Cem. Concr. Compos..

[6] Flatt R.J., Roussel N., Cheeseman C.R. (2012). Concrete: An Eco Material That Needs to Be Improved. J. Eur. Ceram. Soc..

[7] Habbaba A., Lange A., Plank J. (2012). Synthesis and Performance of a Modified Polycarboxylate Dispersant for Concrete Possessing Enhanced Cement Compatibility. J. Appl. Polym. Sci..

[8] Lei L., Plank J. (2014). Synthesis and Properties of a Vinyl Ether-Based Polycarboxylate Superplasticizer for Concrete Possessing Clay Tolerance. Ind. Eng. Chem. Res..

[9] Ran Q., Somasundaran P., Miao C., Liu J., Wu S., Shen J. (2009). Effect of the Length of the Side Chains of Comb-like Copolymer Dispersants on Dispersion and Rheological Properties of Concentrated Cement Suspensions. J. Colloid Interface Sci..

[10] Shu X., Zhao H., Wang X., Zhang Q., Yang Y., Ran Q., Liu J. (2017). Effect of Hydrophobic Units of Polycarboxylate Superplasticizer on the Flow Behavior of Cement Paste. J. Dispers. Sci. Technol..

[11] Malferrari D., Fermani S., Galletti P., Goisis M., Tagliavini E., Falini G. (2013). Shaping Calcite Crystals by Means of Comb Polyelectrolytes Having Neutral Hydrophilic Teeth. Langmuir.

[12] Reese J., Plank J. (2011). Adsorption of Polyelectrolytes on Calcium Carbonate—Which Thermodynamic Parameters Are Driving This Process?. J. Am. Ceram. Soc..

[13] Whitby C.P., Scales P.J., Grieser F., Healy T.W., Kirby G., Lewis J.A., Zukoski C.F. (2003). PAA/PEO Comb Polymer Effects on Rheological Properties and Interparticle Forces in Aqueous Silica Suspensions. J. Colloid Interface Sci..

[14] Uchikawa H., Hanehara S., Sawaki D. (1997). The Role of Steric Repulsive Force in the Dispersion of Cement Particles in Fresh Paste Prepared with Organic Admixture. Cem. Concr. Res..

[15] Yamada K., Takahashi T., Hanehara S., Matsuhisa M. (2000). Effects of the Chemical Structure on the Properties of Polycarboxylate-Type Superplasticizer. Cem. Concr. Res..

[16] Winnefeld F., Becker S., Pakusch J., Götz T. (2007). Effects of the Molecular Architecture of Comb-Shaped Superplasticizers on Their Performance in Cementitious Systems. Cem. Concr. Compos..

[17] Chuang P.-H., Gu Q., Tseng Y.-H., Chen C.-L. (2014). Estimation of Electron Transfer Properties of Ferrocenyl-Dicholesteryl-Peptide in Liquid and Gel. J. Colloid Interface Sci..

[18] Tseng Y.-H., Chuang P.-H., Huang Y.-R., Chen C.-L. (2017). Computational Investigation into the Interactions of Traditional Chinese Medicine Molecules of WenQingYin with GluR2. Int. J. Mol. Sci..

[19] Zhang P., Hou D., Liu Q., Liu Z., Yu J. (2017). Water and Chloride Ions Migration in Porous Cementitious Materials: An Experimental and Molecular Dynamics Investigation. Cem. Concr. Res..

[20] Hirata T., Branicio P., Ye J., Zheng J., Tomike Y., Lange A., Plank J., Sullivan M. (2017). Atomistic Dynamics Simulation to Solve Conformation of Model PCE Superplasticisers in Water and Cement Pore Solution. Adv. Cem. Res..

[21] Zhao H., Wang Y., Yang Y., Shu X., Yan H., Ran Q. (2017). Effect of Hydrophobic Groups on the Adsorption Conformation of Modified Polycarboxylate Superplasticizer Investigated by Molecular Dynamics Simulation. Appl. Surf. Sci..

[22] Hirata T., Ye J., Branicio P., Zheng J., Lange A., Plank J., Sullivan M. (2017). Adsorbed Conformations of PCE Superplasticizers in Cement Pore Solution Unraveled by Molecular Dynamics Simulations. Sci. Rep..

[23] Yamada K., Ogawa S., Hanehara S. (2001). Controlling of the Adsorption and Dispersing Force of Polycarboxylate-Type Superplasticizer by Sulfate Ion Concentration in Aqueous Phase. Cem. Concr. Res..

[24] Shui L., Sun Z., Yang H., Yang X., Ji Y., Luo Q. (2016). Experimental Evidence for a Possible Dispersion Mechanism of Polycarboxylate-Type Superplasticisers. Adv. Cem. Res..

[25] Sun H. (1998). COMPASS:  An Ab Initio Force-Field Optimized for Condensed-Phase ApplicationsOverview with Details on Alkane and Benzene Compounds. J. Phys. Chem. B.

[26] Darden T., York D., Pedersen L. (1993). Particle mesh Ewald: An N⋅log(N) method for Ewald sums in large systems. J. Chem. Phys..

[27] Luo Z., Jiang J. (2012). PH-Sensitive Drug Loading/Releasing in Amphiphilic Copolymer PAE–PEG: Integrating Molecular Dynamics and Dissipative Particle Dynamics Simulations. J. Control. Release.

[28] Eslami H., Karimi-Varzaneh H.A., Müller-Plathe F. (2011). Coarse-Grained Computer Simulation of Nanoconfined Polyamide-6,6. Macromolecules.

[29] Gelardi G., Sanson N., Nagy G., Flatt J.R. (2017). Characterization of Comb-Shaped Copolymers by Multidetection SEC, DLS and SANS. Polymers.

[30] Qian Y., De Schutter G. (2018). Different Effects of NSF and PCE Superplasticizer on Adsorption, Dynamic Yield Stress and Thixotropy of Cement Pastes. Materials.

[31] Mark P., Nilsson L. (2001). Structure and Dynamics of the TIP3P, SPC, and SPC/E Water Models at 298 K. J. Phys. Chem. A.

[32] Pellenq R.J.-M., Kushima A., Shahsavari R., Van Vliet K.J., Buehler M.J., Yip S., Ulm F.-J. (2009). A Realistic Molecular Model of Cement Hydrates. Proc. Natl. Acad. Sci. USA.

